# Editorialets

**Published:** 1907-12

**Authors:** 


### Editorialets.

---

MERRY CHRISTMAS.—The "Red Back" sends greeting to its friends everywhere, and wishes them all A Merry Christmas and great hunks of Prosperity—with a capital P.

---

THE North Texas Medical Association will meet in Fort Worth, December 9th and 10th inst.

---

DR. IRA W. COLLINS, the Osteopath member of the State Board of Medical Examiners for Texas, has resigned.

---

UP AGAINST IT.—An Associated Press dispatch from San Antonio states that at the instance of Dr. V. P. Armstrong—"Assistant State Health Officer"—the doctors of Bexar county have been reported to the grand jury for failure to report births and deaths, as required by law.

---

St. Louis Medical Review.

WHEN this you see, remember me (and the little bill sent you lately), for the north wind doth blow and we shall have snow, and coal is $10 a ton, my son.

---

AND NOW all Alabama is "dry." There is a big movement on to make New Orleans all dry. Let the work go on until the blot on civilization is wiped out all over the world. Selah!

---

DR. ALONZO GARWOOD, of New Braunfels, was unanimously elected President of the San Antonio (Fifth Councilor's) District Medical Association at its late annual meeting in San Antonio.

---

THE BRIEF CHANGES HANDS.—Dr. Lawrence, the founder of the famous *Medical Brief*, and who made a big fortune out of it, now, at the age of 72, retires, and has sold out to Mr. J. H. Strong. *Vale!*

---

MARRIED.—At Decatur, Ill., November 12th ult., Dr. Alonzo Garwood, of New Braunfels, Texas, to Miss Bertha Harpstrite, of Decatur. The "Red Back" extends congratulations and best wishes.

---

ON ACCOUNT of the epidemic of dengue fever prevailing in Monterey at this time, the meeting of the International Medical Association of Mexico, which was to be held in that city on November 18, 19, 20 and 21, has been postponed to January 23, 24, 25, 1908.

---

I HAVE great regard for the "Red Back" for its honesty and fearlessness.

Yours very truly,
C. C. WALKER, M. D.,
Gainesville, Texas.

---

THE latest triumph of science (Professor Crooke) is the manufacture of nitric acid in large quantities, from air. Crooke has commercialized his products. Nitrates (sodium and calcium) are now available in unlimited quantities for fertilizers, and guano and bone dust and all other fertilizers are now simply back-numbers.

---

ALCOHOL AND INSANITY.—Dr. C. L. Gregory, Superintendent of the North Texas Hospital for Insane, has sent in his report to the Governor. It is most interesting, and gives some valuable information, especially as to the relation of alcohol to insanity. In another part of this issue I reproduce an extract on that subject. Read it!

---

JUDGE CLARK BELL, of New York, the distinguished editor of the *Medico-Legal Journal,* and the organizer and first President of the American International Congress on Tuberculosis, and Mrs. Bell celebrated the fiftieth anniversary of their wedding, recently, in New York. On the occasion they were the recipients of many presents and warm congratulations. The JOURNAL wishes them "many happy returns."

---

A GEM FROM INDIANA.—A reader for a New York publishing house gives the following, quoted from a story submitted by an Indiana authoress, as being about the choicest bit he has come across in many years:

"Reginald was bewitched. Never had the baroness seemed to him so beautiful as at this moment, when, in her dumb grief, she hid her face."—*December Lippincott's.*

---

THE MANOR SANITARIUM.—A commodious and well furnished and equipped building, with ample grounds and attractive surroundings, is offered for lease or sale. Correspondence solicited.

A. K. ANDERSON,
JNO. E. HILL,
JOHN E. SELLSTROM,
For Executive Board.

---

"IS THE room disinfected?"

"Yes, mother; and I have sterilized the curtains, deodorized the furniture, asepticized all the fixtures, vaporized the air, washed my lips in an antiseptic solution, and——"

"Have you asepticized the mistletoe?"

"Thoroughly, mother; everything is done. Arthur is waiting now in the hydrogen room."

"Then you may go in and let him kiss you, dear."—*December Lippincott's.*

---

BERI BERI IN TEXAS.—An epidemic of beri beri broke out in the (Austin) State Lunatic Asylum last summer—170 cases; 17

deaths. Because of the drought it was impossible to get fresh vegetables, and the disease was caused by diet largely restricted to rice and other cereals. It is a late discovery that rice causes the disease. Dr. Braddon, an English army surgeon, has recently published a book on the subject, in which he concludes that stale, decorticated rice contains a poison which produces beri beri. It is similar to that fungus on rye—spurred rye—from which we get ergot. I will have more to say on this subject later.

---

AND NOW, Reed & Carnrick's products have fallen under the axe of ye immaculate "Council of Pharmacy," and in a recent publication (*Journal A. M. A.*) they accuse R. & C. of knowingly making false statements. Reed & Carnrick's preparations are rational, and I doubt if the Octopus' anathema will cause many physicians to discontinue their use. I note, by the bye, that a number of ye elect,—I-am-holier-than-thou, ye "unco' gude" satellites of ye Octopus, i. e., the State Association organs—are still carrying R. & C.'s advertisements. Jones should get after them.

N. B.—Reed & Carnrick do not advertise in the "Red Back." More's the pity. Hence I am not "subsidized."

---

AN AUSTIN DOCTOR A SUCCESSFUL INVENTOR.—Dr. J. W. Carhart, of Austin, has invented and has secured patents in America and Europe for an indestructible tire for automobile vehicles. It will entirely supersede the abominable, dangerous and very expensive inflated rubber tires, and the cost is about one-third as much. The tire is of tough wood or paper fiber, mixed with rubber. It can't be punctured. The tire is in use in Paris, France, and Dr. Carhart left on the 21st of November (ult.) for that city to look after his interests. He will be absent two months, and incidentally will attend the clinics and visit the great hospitals of Paris, London, Vienna and Berlin. Dr. Carhart will send us some "notes" for our January number. He is a fine writer.

---

DR. CROTHERS on "Relations of the Doctor to the Alcoholic Problem," T. D. Crothers, M. D., Hartford, Conn. We have received a reprint of this splendid paper—from the *Medical Fortnightly,* St. Louis, October 25, 1907. The paper was read before the Mississippi Valley Medical Association at Columbus, Ohio, October 10, 1907. I wish every physician could read it; it would disabuse their minds of some old and deep-rooted errors as to the effects of alcohol in health and disease. Since the discovery of phagocytosis, it has been demonstrated under the microscope that alcohol inhibits that process by *putting to sleep* the phagocytes—as pointed out by Metchnikoff; thus defeating nature's intelligent efforts at resistance. Dr. Crothers will be pleased to send anyone a copy, free, on receipt of a request to do so.

---

SOME of the most eminent of the foreign investigators in the field of medical research have accepted the invitation of the Committee on Arrangements of the National Convention on Tuberculosis to take part in the series of lectures that will be delivered during the session of Congress in Washington. The convention will be held from September 12 to October 21, 1908, in Washington. The committee has announced the offer of three cash prizes of $1000 each for the first best evidence of effective work in the prevention or relief of tuberculosis done by any voluntary association since the last International Congress in 1905; second, for the best exhibition of a tuberculosis sanitarium for the working classes; third, for the best exhibit of a furnished home for wage-earners.

Several prizes of smaller value are offered for educational leaflets and for special exhibits. The Governors of all the States have been requested to arrange for representation through one or more departments of the State governments.

---

A RARE OPPORTUNITY FOR A CAPABLE DOCTOR.—Texas is rapidly filling up, the vacant lands are being settled, building of towns and railroads is on a boom, real estate of all kinds is increasing in value, especially farming lands. Now is the time to get in "on the ground floor." In Ellis county (Central North Texas), in the very heart of the rich black alluvial lands (bale-to-the-acre land), there is offered a doctor's home, complete with all requisites, residence, outhouses, three lots of ground, orchard, garden, wells; a new buggy and pair of matched bays (double or single harness or saddle), worth $450; a $600 stock of drugs and an established and secure practice netting $3000 a year, all for $2500; $2000 cash, balance to suit. It is in a thriving town of 1500; accessible from all points by rail. The owner is not in robust health and wants to go to a city to practice (indoors) a specialty. He guarantees to induct his successor into the practice; turn it over to him, so that the purchaser will have nothing to do but move in with his family and hang up his hat; step into a ready-made home and good practice. It takes years to get fixed and build a practice. The property alone is worth double the price asked for the whole. Write to me about it.

EDITOR TEXAS MEDICAL JOURNAL.

